# Psychological Disturbances and Quality of Life in Obese and Infertile Women and Men

**DOI:** 10.1155/2012/236217

**Published:** 2012-07-16

**Authors:** Piotr Kocełak, Jerzy Chudek, Beata Naworska, Monika Bąk-Sosnowska, Barbara Kotlarz, Monika Mazurek, Paweł Madej, Violetta Skrzypulec-Plinta, Piotr Skałba, Magdalena Olszanecka-Glinianowicz

**Affiliations:** ^1^Health Promotion and Obesity Management Unit, Department of Pathophysiology, Medical University of Silesia, 40-752 Katowice, Poland; ^2^Pathophysiology Unit, Department of Pathophysiology, Medical University of Silesia, 40-752 Katowice, Poland; ^3^Obstetric Propaedeutics Unit, Department of Woman's Health Care, Medical University of Silesia, 40-752 Katowice, Poland; ^4^Psychology Unit, Medical University of Silesia, 40-752 Katowice, Poland; ^5^Department of Endocrinological Gynecology, Medical University of Silesia, 40-752 Katowice, Poland; ^6^Department of Women's Health, Medical University of Silesia, 40-752 Katowice, Poland

## Abstract

Anovulatory cycles and endometriosis are the main causes of female infertility. The most frequently anovulatory cycles are related to polycystic ovary syndrome (PCOS) commonly associated with obesity and hormonal disturbances in the course of obesity. Recently published studies revealed that infertility affects about one in six couples during their lifetime and is more frequent in obese. Obesity is also associated with male infertility related to erectile dysfunction, hormonal disturbances and lower semen quality. Any of these above mentioned disorder is the important risk factor of psychological disturbances and poor quality of life among women and men in the reproductive age. On the other hand the mood disorders may exacerbate the hormonal disturbances and worsen the effectiveness of infertility management. Infertility, its therapy with accompanying psychological disturbances may also significantly affect the partners relationships. The review summarize the results described in the current literature on the association between obesity and infertility and psychological disturbances as well as their impact on quality of life and sexual functioning in women and men. Moreover, the impact of infertility and psychological disturbances on partners relationships is discussed.

## 1. Introduction

The number of infertile subjects worldwide in 2005 year was estimated at 60–80 million with their annual growth on about 2 million. In developed countries infertility is diagnosed in 17–26% of reproductive age couples. The prevalence of infertility increases with age from 20% among subjects 35–39 years old to 25–30% among those 40 years and over [1]. The percentage of female and male infertility causes is similar (40%), and in 20% of couples both partners are affected [2].

Infertility is defined as inability to conceive a child by a couple in a stable relationship during the year of regular intercourse without the use of contraceptive methods.

The prevalence of obesity in Europe is estimated at 10–20% of men and 10–25% of women [3], while in the United States of America at 32.2% and 35.5% [4], respectively, with continuous tendency to grow. Thus in consequence the number of men and women diagnosed with infertility related to obesity is also increasing [5, 6].

Anovulatory cycles and endometriosis are the main causes of female infertility. The most frequent anovulatory cycles are related to polycystic ovary syndrome (PCOS) occurrence, commonly associated with obesity and hormonal disturbances in the course of obesity [7, 8]. Recently published studies revealed that infertility affects about one in six couples during their lifetime and is more frequent in obese [9]. The prevalence of PCOS is estimated at 5–10% of women in childbearing age. Hormonal disturbances in PCOS include insulin resistance, hyperinsulinemia, inadequate gonadotropins secretion, and hyperandrogenism [10]. In the last decade the results of numerous studies revealed that hormones of adipose tissue (adipokines) play a role in the PCOS development [11, 12]. Infiltration of adipose tissue with macrophages, disturbed adipokines secretion, increased lipogenesis, and free fatty acids release constitute the key elements in the pathogenesis of insulin resistance development [13, 14]. Moreover, adipokines may participate in the PCOS development by other pathways. It has been suggested that changes of their secretion influence LH and FSH release as well as directly affect ovary steroidogenesis [11, 12, 15, 16], while PCOS-related hyperandrogenism manifests clinically by irregular menstruation, hirsutism, acne, and hair loss and frequently by infertility [17].

The risk factors of male infertility include age, some chronic diseases, especially obesity and its related disorders as well as infectious diseases, use of some medications, environmental factors (lead, arsenic, aniline dyes, ionizing radiation, electromagnetic fields, exposure), and lifestyle factors (high-fat and high-caloric diet, low physical activity, smoking, drinking and drug use, as well as tight and plastic clothing) [5, 6].

The relationship between obesity and infertility in men was first described by Avicenna in the 10th century. Current studies revealed that the risk of infertility increases with obesity grade [18] regardless of age and female partner's BMI and smoking habits of both partners [19]. It has also been shown that obese couples where both partners are affected are less fertile than those with normal body mass [18, 19].

It is well known that obesity is associated with erectile dysfunction. The risk factors of erectile dysfunction include obesity grade, visceral obesity, low testosterone level, and physical inactivity. The pathophysiological links between obesity and erectile dysfunction are poorly understood. It is suggested that the relationships include endothelial dysfunction, especially decreased endothelial nitric oxide synthase (eNOS) activity and NO release related to chronic, systemic microinflammation and insulin resistance as well as suppression of hypothalamic-pituitary-testicular axis. It has also been shown that decreased testosterone level in obese men increases the risk of vascular pathology occurrence. Thus, both testosterone deficiency and endothelial dysfunctions related to the other disturbances are factors causing the decreased eNOS expression and activity followed by penile vascular insufficiency. Currently, it is established that erectile dysfunction is the symptoms of endothelial injury. On the other hand, as it was mentioned above, testosterone deficiency is a risk factor of endothelial dysfunction and cardiovascular disease development [20, 21]. Pasquali et al. [22] showed that weight loss improves androgen imbalance and erectile function.

 Obesity-related hormonal disturbances are not restricted to androgen deficiency. It was suggested that decreased sex hormone-binding globulin (SHBG) and increased free testosterone levels in consequence favor testosterone to estradiol conversion in adipose tissue. Decreased testosterone-to-estradiol ratio contributes to impaired spermatogenesis and infertility development [23]. Regardless of the lower total testosterone level, its free fraction is decreased only by 5% in obese than in normal weight men due to markedly lower levels of circulating SHBG [24]. The decrease of SHBG synthesis is related to insulin resistance. It should be emphasized that hormonal disturbances worsen proportionally to the degree of obesity [25]. It has been shown that testosterone level in obese men is inversely proportionally related to circulating leptin concentration. Leptin directly inhibits testosterone synthesis by membrane receptors on testicular Leydig cells [26–28]. Additionally, decreased LH and inhibin B levels in obese young men were found [29, 30]. Moreover, the results of experimental study revealed that inhibin B level is the marker of Sertoli cells number [31]. Thus decreased inhibin B level in obese men may be associated with fewer number of Sertoli cells and lower sperm count. It has also been suggested that decreased insulin-like factor 3 level in obese men is the result of a primary Leydig cells dysfunction and may be reliable marker of this cells general impairment [32]. The results of studies assessing the impact of obesity or visceral obesity on semen parameters are inconsistent. Some [23–35] but not all studies [36–38] revealed negative relation between BMI and sperm parameters.

Both obesity and infertility are the important risk factors of psychological disturbances and poor quality of life among women and men in reproductive age. On the other hand the mood disorders may exacerbate the hormonal disturbances and worsen the effectiveness of infertility management.

Female gender *per se* is the risk factor for mood disorders development. The prevalence of mood disorders, especially major depression and anxiety in general population, is higher by 50% among childbearing age women than in men [39–41]. The occurrence of mental illness is significantly increased among infertile subjects [42].

The paper summarizes the results described in the current literature on the association between obesity and infertility and psychological disturbances as well as their impact on quality of life and sexual functioning in women and men. Moreover, the impact of infertility and psychological disturbances on partners relationships is discussed.

## 2. The Possible Links between Obesity and ****Depression

Disturbances in serotonin release are the cause not only of mood decrease but also increase in consumption of carbohydrate-rich foods and aversion to physical activity [43]. Additionally, stress and depression symptoms are associated with increased hypothalamic-pituitary-adrenal (HPA) axis activity. In turn, its chronic overactivation and excessive cortisol level may be responsible for fat accumulation, especially of visceral localisation. It is suggested that brain reward circuitry plays the central role in the activation of stress-induced food intake. Furthermore, highly palatable food or activation of HPA axis releases opioids attenuating detrimental effect of stress response by inhibiting HPA axis. Moreover, elevated cortisol level increases food consumption by changes in action of mediators regulating hunger and satiety, such as neuropeptide Y (NPY), leptin, and insulin [44]. The results of a single study revealed that chronic microinflammation related to obesity may be a factor that participates in depression development [45]. However, our study did not confirm this link between obesity and depression [46]. The results of experimental studies have also suggested that gastrointestinal tract hormones, such as ghrelin and peptide YY (PYY), participate in mood disorders development. It was shown that in rats under chronic stress condition increased ghrelin levels defend against depressive symptoms [47]. The mechanism of ghrelin action on mood is associated with the suppression of cannabinoid-1 (CB1) and melanin-concentrating hormone-1 receptors by this hormone [48, 49]. In turn the results of clinical studies revealed that suppression of CB1 receptors by its antagonist had the increased potential for depression development in human [50]. On the other hand ghrelin increases the expression of NPY in hypothalamic neurons and NPY has antidepressant activity, in both rodents and human [51], while PYY inhibits the release of NPY [52]. In obese subjects decreased circulating levels of both ghrelin and PYY were shown [53, 54], and weight loss was increasing their levels [55, 56]. However, we did not observe differences between plasma ghrelin and PYY levels in obese subjects with and without depression [57]. As shown above pathophysiological links between obesity and mood disorders are multifactorial and not fully clarified. The results of previously published studies indicated the mutual stimulation of both diseases in their development. Furthermore, changes in the activity of HPA axis related to depression may contribute to the occurrence of infertility, enhanced by the changes of hormonal adipose tissue activity in obesity.

## 3. Mood Disorders in Obese and Infertile ****Women

The epidemiological studies revealed higher prevalence of mood disorders, especially depression, in obese women (14% versus 10%, resp.) but not in obese men, than in the general population [58–61]. Of interest obese men had lower risk of major depression [60].

 It should be also emphasized that the prevalence and severity of depression symptoms among obese subjects referred both to a conservative and surgical treatment with a degree of obesity [62–65]. It is suggested that the overrepresentation of depressive subjects among obese women is associated with binge eating disorder [66].

Mood disorders in obese may by partially related to low self-esteem. Social discrimination of obese starting from childhood, assigning them negative features of character and self-awareness of lower attractivity, is more pronounced in women [67]. Thus women have lower self-esteem, which predisposes them to mood disorders development [68]. Morbid obesity is associated with the highest discrimination, comorbidity, and the lowest health-related quality of life, as well as the frequent tendency for food intake in response to negative emotions [69]. Recently an association between body mass and distress has also been found [70]. Other risk factors for depression symptoms development in obese are chronic bodily pain, disability, self-care, and work-related activities difficulties as well as stigmatization of obese subjects [71].

It has also been shown that lifetime diagnosis of depression and anxiety is associated with obesity [72]. The risk of depression development in obese American aged 50 years or over during 5-year followup was doubled [73].

On the other hand, it was shown that depression is also a risk factor of obesity development during 2-year followup [74]. Additionally, in adolescent girls depression was a predictor of obesity development, while obesity was the risk factor of depressive episodes number [75]. The factors favoring obesity development in depressive subject are lower physical activity and increased hunger related to the use of some psychotropic drugs [76]. Additionally, depression predicts visceral fat accumulation associated with higher activity of HPA axis and adrenal gland volume as well as corticosteroids levels [77–79]. The significant association between major and moderate depressive symptoms and waist circumference is independent from the coexistence of chronic diseases, including overweight and obesity [80, 81].

Weight loss above 5% of the initial body mass improves mood, bodily satisfaction, self-esteem, and self-confidence as well as physical health [82–86]. It has also been shown that the occurrence of psychological disturbances, especially depression, anxiety, and binge eating does not predict the adherence and effectiveness of weight loss programs [87]. However, mood disorders may disturb the compliance to dietary and physical activity recommendations [88].

It is doubtful whether the weight reduction itself may significantly improve the course of severe depression [88]. Therefore obese with severe depression necessitate pharmacotherapy and psychotherapy before and during weight loss management [89, 90]. There is the lack of studies assessing the impact of mood disturbances on weight maintenance as well as the weight regain consequences on mood disorders.

The prevalence of anxiety among obese subject is estimated at 37%, and unlike depression, it is not associated with gender, education level, and treatment referral [91]. It seems that occurrence of anxiety is more persistent than depression symptoms. Anxiety seems to be a trigger for emotional eating as well as for bingeing. The results of numerous studies revealed an association between anxiety or emotional eating and obesity [92].

The associations between mental health in PCOS women and age, BMI, education level, physical activity and infertility were found [93]. Some authors showed that both negative body image and self-worth are important risk factors of depression and anxiety development in PCOS women as well as decreased QoL [94]. In PCOS women, obesity is the main risk factor for development of mental illness, anxiety, and social fear [95]. A higher prevalence and severity of depression has been reported in PCOS women [96, 97]. It is related to obesity, emotional disturbances, hirsutism, acne, menstrual predictability, and infertility [96]. Obesity interferes with self-perception and decreased self-esteem as well as impairs sexual functioning. Infertility may be a cause of misunderstandings and tension in the family and generate problems in the workplace, especially in women who had never given birth [98, 99]. Adolescent girls diagnosed with PCOS report obesity as most distressing symptom of the disease. Additionally, the relationship between self-perception and depression level was shown [100]. The disturbed self-perception in adolescent PCOS is an often cause of extreme exercise application for weight control and eating disorders development [101]. Moreover, in these girls self-esteem is also decreased by acne and hirsutism [102], while the adult PCOS women have less feminine self-perception due to infertility, hirsutism, and menstrual problems [103]. The association between hirsutism degree and anxiety, psychotic symptoms sadness and frustration was found [94, 104]. Of interest the results of a single study showed beneficial effect of higher free testosterone level on mood [105]. However, other studies did not reveal the association between depression and testosterone level [96]. The associations between depressive disorders or social phobias and BMI as well as free androgen index (FAI) values in PCOS women were also found [106]. It was also suggested that the elevated LH and androgens levels which alter the monoamine balance are the link between mood disorders and PCOS [107], while some authors claim a positive relationship between depressive symptoms and HOMA-IR values but not irregular menses, hirsutism, hair loss, or acne [95].

In PCOS group frequent suicide attempts as well as anxiolytic and antidepressant drugs use were reported [101]. On the other hand depression occurrence may exacerbate the hormonal disturbances in PCOS women, while its treatment may ameliorate these symptoms [96, 107]. The lower prevalence of depression was found among PCOS women receiving oral contraception [95]. It is interesting observation because oral contraception given for contraceptive purposes increases frequency of depression incidence itself [108].

As it was mentioned above hormonal disturbances occurring in obese and PCOS women are common causes of infertility, and both these conditions are associated with higher prevalence of depression symptoms. Additionally, infertility increases the risk of mood disorders development.

Numerous studies showed that more than 50% of couples consider infertility as the most disappointing experience in their life [109]. Even 80% thought about infertility as stressful or very stressful experience [110] or as feeling of grievance, depression, sin, threat, marital problems, and disappointment [111]. On the basis of the results obtained by self-reported Beck Depression Inventory (BDI) the depression symptoms were shown in 37% of infertile women; among them 19.4% have moderate or severe depression symptoms [112, 113]. Many factors such as causes and length of infertility and the therapeutic strategies may influence psychological disturbances in infertile couples in a different way among men and women [114]. It is suggested that depression development in infertile women is the result of prolonged and severe normal psychological response to the diagnosis which is the grief and mourning [115]. It has also been found that the frequency of depression in infertile women increases with the duration of therapy [116].

Higher frequency of mood disorders (anxiety and depression) was shown in infertile women undergoing *in vitro* fertilization (IVF), especially during embryo transfer or egg retrieval [114]. Before initiation of infertility treatment, depression was diagnosed in 33% of women and 3.5% of men, and its prevalence increased after IVF failure to 43% and 8%, respectively [117, 118]. At the same time, the frequency of anxiety increased from 10.6% before to 14.2% after IVF in women [118]. Women are more prone to be affected by stress, anxiety, and discouragement and are more engaged into the diagnostic and therapeutic procedures [45]. Moreover, the risk factors of mood disorders in infertile women are time of applying for pregnancy, younger age, and partner with anxiety symptoms. In contrast, the risk factors of mood disorders in infertile men or partners of infertile women include temporary job, the first visit of IVF treatment, and an anxious partner [119]. However, the results of another study did not confirm the influence of diagnosis, age of female, duration of infertility, number of IVF procedures on mood disorders in infertile women and revealed that mood disorders are affected by personality and stress coping strategies [20]. The absence of support from husband and social discrimination are among the important risk factors of mood disorders development and main stressors disturbing GnRH pulsatility resulting in anovulatory cycles in infertile women [120]. It should be emphasized that psychological distress during fertilization and pregnancy may increase the rate of complications in the newborn at the time of and after delivery. Mothers conceived by IVF are more worried about survival and normality of their unborn babies, about damage to their babies during childbirth, and about separating from their babies after birth [121].

Depressive symptoms are associated with menstrual abnormalities, such as rare menarche, irregular cycles, and secondary amenorrhea [122]. The relationship between menstrual cycle disturbances and mood disorders seems to be reciprocal. The prevalence of depressive symptoms in women with functional hypothalamic amenorrhea (FHA) is similar to that observed in women with organic menstrual abnormalities [123]. The lower rates of fertility have been reported in women with a history of major depression episodes [124]. However, one study restricts the lower rate of fertility only to depressive women with suicidal attempts [125].

The impaired fertility in depressive women may be also related to the use of antidepressants responsible for the higher risk of spontaneous abortion, hyperprolactinemia, hyperandrogenism, hyperinsulinemia, and menstrual disturbances [126].

The explanation of the association between mental illness and infertility is difficult. It is caused by the different definitions of infertility, length of marriage attempts to conceive and infertility treatment timing of the diagnosis (before or after the onset of psychiatric episode), age, as well as other causes of decreased fertility such as male infertility, cultural factors and availability of assisted conception [127–129].

It was revealed that development of somatization, depression, anxiety, and paranoid ideation is associated with actual treatment for infertility and feelings of loneliness as well as sexual and financial factors [130].

Psychotherapy of infertile couples decreases anxiety and depression resulting in improvement of HPA axis function and increases the likelihood of conception [129, 130]. It has also been shown that isolated psychological interventions increase the rate of pregnancy [131]. Successful conception is reported to be lower in women with the history of infertility if followed by a high rate of major depression (69.2% versus 30%), anxiety (23.1% versus 3%), panic disorders (15.4% versus 0%), phobia (23.1% versus 10%), and bulimia (7.7% versus 0%) during pregnancy [132]. Thus, the results of this study necessitate psychological care in this group of pregnant women.

## 4. Other Psychological Disturbances and Mental Illness in Obese and Infertile Women

The higher prevalence of distress, interpersonal sensitivity, and obsessive-compulsive symptoms has been shown in PCOS women [133, 134], while similar anger and aggression levels in PCOS and non-PCOS women were found [94]. Additionally, body dissatisfaction related to hirsutism and negative body image self-perception are more frequent among PCOS women [94, 133]. Body dissatisfaction in adolescents may be a risk factor of eating disorders and depression development [135]. However, the results of studies that assessed the prevalence of eating disorders in PCOS women are inconsistent. Some studies [136], but not all [137], revealed higher frequency of bulimia in PCOS women. On the other hand, it is suggested that bulimia is the risk factor of PCOS development as overeating and starving episodes impair insulin sensitivity and are associated with ovarian morphology changes [136].

Mental illness is more frequently diagnosed in infertile women than men (61.1% versus 21%). The most prevalent disturbances among infertile subjects are mood disorders (59.6%), especially anxiety (67% infertile women). Dysthymia, somatization, and conversion disorders were observed in infertile women only. The risk factors of psychiatric morbidity among infertile subjects include female gender, the quality of the couple's relationship, type and length of infertility as well as diagnostic procedures, number of treatment cycles, and outcome of IVF [45, 138]. A great need for conceiving a child among PCOS than non-PCOS women (42% versus 6%) is considered as the important risk factor of psychological and emotional disturbances [134].

The higher depression, psychoticism, and somatization levels and worse interpersonal relations were found among infertile than fertile women [21], while, the results of other studies revealed higher prevalence of paranoid ideation and lower psychoticism and phobic anxiety in infertile women [129]. Additionally, dominance of somatization and anxiety and lower occurrence of psychoticism and panic phobia among these subjects have also been shown [139].

The higher prevalence of alexithymia in infertile women was found. Alexithymia seems to be the psychological response and the strategy of coping as well as adaptation to the diagnosis and the infertility management [140]. It is also considered as the form of depression [141].

## 5. Quality of Life (QoL) in Obese and Infertile ****Women

Obesity decreases both physical and mental aspects of quality of life, especially in women [142]. Health-related quality of life (HRQoL) decreases proportionally to increased BMI and fluctuations in body weight, coexisting comorbidities including mental illness and binge eating. The physical aspect of HRQoL is deteriorated especially by the coexistence of mood disorders, obesity, and its comorbidities [31]. Among subjects aged 60 years or more, obesity is associated with worse physical functioning regardless of age, especially in women [143]. Among elderly population higher waist circumference is related to lower HRQoL but higher occurrence of depressive symptoms only in women [144]. Surprisingly, higher HRQoL in obese men was found [145]. Additionally, it is shown that physical domain of HRQoL diminishes, while self-esteem and public distress improve with age [144]. Obesity decreases self-esteem and increases body dissatisfaction especially in adolescents and young women. Low self-esteem particularly coexisting with anxiety is the reason of social isolation among obese subjects [146]. Elevated BMI and waist circumference values are independent risk factors of urogenital dysfunction, especially urinary incontinence which is the reason for decreasing mostly emotional aspect of HRQoL and increasing feeling of frustration but not affect sexual lust [147, 148]. It should be emphasized that BMI affects the HRQoL independently of questionnaire used to its assessment [149]. In morbid obese significant improvement of HRQoL, both physical and psychological aspects as well as self-esteem, social life, sexual activity, and disposition for physical activities or work, after surgical treatment of obesity with large weight loss were obtained [150–152]. Regardless of the observed improvement in HRQoL and disease-specific quality of life (DSQoL) one-year after procedure and their maintenance during 5-year followup the HRQoL values are lower than in age- and gender-matched general population [153].

All physical, psychological, and emotional aspects of HRQoL are decreased [136] and strongly associated with overweight/obesity and hirsutism grade in PCOS women [97]. It was found that in PCOS women weight loss obtained by pharmacotherapy decrease in hirsutism severity improves mostly psychological domain of HRQoL [154]. Additionally, infertility seems to be important factor that alters psychological aspect of HRQoL in PCOS women [155]; however duration and the cause of infertility seem not to influence HRQoL [156]. Among couples undergoing IVF procedure higher HRQoL was shown in males than in females [157]. However, some previously published studies did not confirm the difference in mental health between infertile and fertile PCOS women [158]. Of interest, higher depression level and lower body satisfaction were found in infertile PCOS than in women infertile due to other causes [133]. Ethnic origin is an important confounder in QoL comparison however in both Caucasian and South Asian PCOS HRQoL was similar and significantly lower than in non-PCOS women. In both ethnic groups obesity and infertility were important factors deteriorating HRQoL, while menstrual disturbances were affecting only Asian PCOS women [159].

It should be emphasized that physical dimension of HRQoL in PCOS women is similar to that observed in patients diagnosed with asthma, epilepsy, diabetes, and back pain, while the mental health is about 20% worse in PCOS [160].

## 6. Sexual Dysfunction and Sexual Aspect of Quality of Life in Obese and Infertile Women

Obesity is associated with fewer sexual partners in both men and women and having a partner at all by women. However, frequency of sexual intercourses and sexual practices is similar in obese and normal weight subjects [161]. Both abortion and unintended pregnancies due to the use of less effective contraceptive methods are more frequently reported in obese women [162].

Obese men have higher tendency to choose nonobese partners than obese women. Stronger social pressure to have a partner for a woman than for a man seems to be the explanation [163].

Some studies revealed that obesity is not associated with sexual dissatisfaction or with experience of sexual abuse. However, it was shown that sexual abuse influences eating behavior and may be the cause for both obesity and anorexia nervosa development [143].

Sexual dysfunction related to obesity was observed in both perimenopausal and postmenopausal obese women with lower HRQoL. It decreased appearance and sexuality self-perception [160]. However, the results of studies assessing the impact of obesity on female sexual dysfunction including arousal, lubrication, orgasm, and satisfaction are inconsistent [164, 165]. Obese subjects frequently report sexual disturbances such as lack of enjoyment of sexual activity, difficulties with sexual performance, lack of sexual desire, and avoidance of sexual encounters. Sexual QoL is mostly impaired in morbid obese women seeking bariatric surgery [166].

An association between worse HRQoL and decreased sexual relationship within marriage, proportionally to hirsutism severity, was reported [167]. It was also shown that HRQoL in PCOS women is affected by emotional problems, pain, energy/fatigue, emotional well-being, and social functioning but not BMI [135]. Nevertheless, the results of other studies showed that obesity is the main factor affecting sexual dissatisfaction and QoL [160]. However, no association between sexual functioning and obesity or infertility as well as androgen levels was found [168].

In PCOS women lower total QoL and sexual satisfaction frequently coexist with anxiety, depressive and obsessive-compulsive disorders [135]. Additionally, worse sexual function is associated with higher androgens and LH levels in PCOS [169]. It has also been found that PCOS women experience less sexual attractiveness and sexual desire [170]. Contrary, the rate of having a sexual partner, the frequency of sexual intercourse or sexual thoughts and fantasies are similar both in PCOS and non-PCOS women. The reason for lower sexual satisfaction may be the imaging that partner felt less satisfied with their sexual life and that he found her as less sexually attractive [136]. Among the factors that influence self-esteem are also obesity, acne, hirsutism, infertility, and psychological distress [135, 170, 171].

## 7. Psychological Disturbances Related to Obesity and Infertility in Men

Only few studies described an association between emotional state and infertility in men. It was shown that acute stress associated, for example, with earthquake and war affects male fertility, not only in the aspect of erectile function but also the quantity and quality of semen [172, 173]. Moreover, the negative effect on semen quality of less traumatic events such as exams has also been found [174]. Additionally, temporary decrease of men fertility related to chronic stress after death of relative was described [175]. It was shown that during stress superoxide dismutase activity increases significantly while catalase activities remain unchanged. Moreover, spermatozoa concentration, motility index, and percentage of rapid progressive motility decrease under stress [176].

It was shown that the reference of a couple to infertility management clinic due to his partner's infertility is related to severe negative emotions in men. Moreover, it was suggested that emotional stress is the frequent risk factor of therapy discontinuation [176, 177].

The above-mentioned negative emotions may be the cause of erectile and ejaculation dysfunction as well as decreased libido and number of relations [178]. On the other hand male reason of infertility may be the cause of guilt or sadness and in turn depression development [179–181]. However, as it was mentioned above the main risk factors associated with development of depression related to infertility are female gender [182]. The occurrence of depression or anxiety symptoms and lower testosterone level impairs spermatogenesis and decreases their sperm concentration [178, 183]. However, another study revealed that sperm count is not associated with anxiety and depression levels, but sperm motility is weakly and inversely related to depression level [184].

The prevalence of depression among infertile men is estimated at 5–15% and is significantly lower than among women [175, 180, 182] and higher in Middle East region than in Western countries [118, 185].

In some countries, especially in Middle East region fatherhood is traditionally sociocultural determinant of masculinity. However, not only in these societies the stigma of infertility in men may be a cause of low self-esteem, considering themselves as second-class men, and reduced sense of masculinity [186].

High scores on neuroticism-related personality traits are risk factors of depression and anxiety disorders development in both infertile male and female. Additionally, high neuroticism scores are negatively associated with live birth [187].

The links between obesity, mood disorders, and infertility in men are poorly understood. Some studies revealed that obese men had lower frequency of depression symptoms [188]. The depression may be a cause of obesity development in men as negative emotions are frequently compensated by compulsive overeating. For example sweets increase serotonin release in brain and decrease tension, give a feeling of pleasure, and improve well-being [189–191]. In men the lack of sense of overeating but not the lack of body size is the cause of decreased mood and life satisfaction [192]. Thus, as was described above depression may be the cause of decreased fertility *per se* and indirectly by stimulation of obesity development. On the other hand in some cases male infertility may be the cause of depression and obesity development. In turn obesity may be the cause of infertility and subsequently development of depression. It should be emphasized that erectile dysfunction related to obesity may be the important risk factor of depression and anxiety development in men. It was found that male diagnosed with new onset erectile dysfunction also reported both depression and anxiety [193]. Moreover, Conrad et al. [194] revealed that some men may experience sexual dysfunction of a psychogenic nature in response to the diagnosis of infertility. It was also suggested that alexithymia and somatization are consequences of coping with male infertility [195]. Alexithymia in infertile men may play a defensive role as far as depression is concerned but increases the possibility of somatic complaints [196]. Regardless which of these disorders is the cause and which is the effect, their coexistence should be considered, and psychotherapy should be important element of this diseases management.

It should be mentioned that a frequent side effect of both typical, but also of many atypical antipsychotics such as amisulpride, risperidone, or ziprasidone used in some mental illness, is hyperprolactinaemia and in turn hypogonadism in both male and female [197]. The interesting observation is that male offspring of patients with schizophrenia had 12% fewer offspring [198].

## 8. Quality of Life and Its Sexual Aspect in ****Obese and Infertile Men

The lower HRQoL in physical domain and social functioning was reported in overweight adolescents. While in obese adolescents, additionally, emotional functioning was decreased. It should be emphasized that excess weight worsened HRQoL in the greater extent in girls than in boys [199]. Similar results were obtained in morbid obese subjects. Women had significantly lower HRQoL level than men, although they were younger, less obese, and less likely to be married. Both in men and in women increasing number of comorbidities was decreasing physical and mental HRQoL [189]. It has also been shown that mental HRQoL was affected by the presence of depression and eating behavior disorders but not by physical comorbidities and obesity grade. However only in men low physical self-perceived well-being was associated with obesity grade [200]. The most important predictor of physical and mental HRQoL components in morbid obese subjects was employment regardless of gender [201, 202]. Among other factors that deteriorated HRQoL in obese of both genders was depression [202]. Subthreshold depression and anxiety were the major predictors of QoL level in infertile men [203]. Isolated male infertility was associated with lower sexual, personal, and social strains QoL in men [204].

## 9. Partners Relationships

The studies assessing the QoL, sexual quality of life, and partnerships in infertile couples revealed interesting results. Shindel et al. [205] show significant discrepancies between partners only in psychological and social relationships domains. Depression in males affected all QoL domains, while in female only overall, psychological and physical. Additionally, in infertile couples male partner sexual function was related to female partner sexual function. The important predictors of men's relationship status in self-assessment were relationship duration and female partner assessment of relationships [206]. Moreover, Elia et al. [207] observed that in infertile couples women had poor marital adjustment QoL. The forced timing of intercourse around the woman's ovulatory cycle is associated with psychological pressure to try to conceive, and men may experience less intercourse satisfaction. Some authors observed that stressful situation related to the diagnosis of subfertility can reduce the pleasure of sex, independently from the severity of the semen alterations awareness [208]. On the other hand it was shown that successfully assisted reproduction technique does not constitute a risk for marital adjustment. Moreover, the shared stress of infertility may even stabilize marital relationships [209]. Additionally, it was found that diagnosis of male factor and infertility duration of 3–6 years were associated with the highest relationship instability and the lowest sexual satisfaction both in infertile females and males [210]. Sand et al. [211] revealed that in infertile couples most common problems are psychosexual disorders, including dyspareunia decreased libido and orgasmic failure in women, and premature ejaculations followed by erectile dysfunction, decreased libido and orgasmic failure in men. Psychosexual dysfunction and infertility were found to occur, in a large number of couples, together in association. These authors suggested that the most common cause for this problem is ignorance and lack of sex education.

Interesting findings were obtained in multinational Men's Attitudes to Life Events and Sexuality (MALES) study. The important determinants of self-perceived masculinity were being seen as honorable, self-reliant, and respected by friends, while factors stereotypically associated with masculinity, such as being physically attractive, sexually active, and successful with women, were deemed to be less important. Moreover, men deemed of significant importance for QoL factors such as good health, harmonious family life, and a good relationship with their wife/partner, while factors such as having a good job, having a nice home, living life to the full, or having a satisfying sex life were far less important. It should be emphasized that these determinants of self-perceived masculinity and QoL were similar in men with or without erectile dysfunction [212]. On the other hand it was also shown that network positions that afford independence and control over social resources are consistent with traditional masculine roles and may therefore affect men's sexual performance. When heterosexual man's female partner has more frequent contact with his confidants than he does—partner betweenness—his relational autonomy, privacy, and control are constrained; men are more likely to report erectile dysfunction [212]. These data suggest that psychological factors and partner relationships are important factors formative of a total and sexual QoL.

In summary, the reviewed data confirm the adverse impact of both obesity and impaired fertility on psychological disturbances and QoL especially in women. Mood disorders exacerbate hormonal disturbances, resulting in anovulatory menstrual cycles and decreased conception. In men mood disorders also decrease fertility by exacerbating erectile dysfunction and decrease semen quality. Mood disorders in both female and male are a risk factor of eating disorders and obesity development. Infertility and mood disorders affect the partners' relationships. Therefore the psychotherapy should be included in the management of both obesity and infertility. Such an approach will improve the effectiveness of infertility therapy and prevent development or worsening mood disorders in obese infertile women and men. 
